# PAK4-NAMPT Dual Inhibition as a Novel Strategy for Therapy Resistant Pancreatic Neuroendocrine Tumors

**DOI:** 10.3390/cancers11121902

**Published:** 2019-11-29

**Authors:** Gabriel Mpilla, Amro Aboukameel, Irfana Muqbil, Steve Kim, Rafic Beydoun, Philip A. Philip, Ramzi M. Mohammad, Mandana Kamgar, Vinod Shidham, William Senapedis, Erkan Baloglu, Jing Li, Gregory Dyson, Yue Xue, Bassel El-Rayes, Asfar S. Azmi

**Affiliations:** 1 Department of Oncology, Wayne State University School of Medicine, Detroit, MI 48201, USA; mpillag@karmanos.org (G.M.); kameelo@karmanos.org (A.A.); kims@karmanos.org (S.K.); philipp@karmanos.org (P.A.P.); mohammad@karmanos.org (R.M.M.); mkamgar@mcw.edu (M.K.); bb8374@wayne.edu (J.L.); dysong@karmanos.org (G.D.); 2 University of Detroit Mercy, Detroit, MI 48201, USA; muqbilir@udmercy.edu; 3 Department of Pathology, Wayne State University School of Medicine, Detroit, MI 48201, USA; rbeydou@med.wayne.edu (R.B.); vshidham@med.wayne.edu (V.S.); 4 Karyopharm Therapeutics Inc., Newton, MA 02459, USA; william@karyopharm.com (W.S.); erkan@karyopharm.com (E.B.); 5 Winship Cancer Institute, Emory University, Atlanta, GA 30322, USA; yue.xue@northwestern.edu (Y.X.); bassel.el-rayes@emoryhealthcare.org (B.E.-R.)

**Keywords:** PAK4, NAMPT, mTOR, pancreatic neuroendocrine tumors, KPT-9274

## Abstract

Pancreatic neuroendocrine tumors (PNET) remain an unmet clinical need. In this study, we show that targeting both nicotinamide phosphoribosyltransferase (NAMPT) and p21-activated kinase 4 (PAK4) could become a synthetic lethal strategy for PNET. The expression of PAK4 and NAMPT was found to be higher in PNET tissue compared to normal cells. PAK4-NAMPT dual RNAi suppressed proliferation of PNET cell lines. Treatment with KPT-9274 (currently in a Phase I trial or analogs, PF3758309 (the PAK4 selective inhibitor) or FK866 (the NAMPT inhibitor)) suppressed the growth of PNET cell lines and synergized with the mammalian target of rapamycin (mTOR) inhibitors everolimus and INK-128. Molecular analysis of the combination treatment showed down-regulation of known everolimus resistance drivers. KPT-9274 suppressed NAD pool and ATP levels in PNET cell lines. Metabolomic profiling showed a statistically significant alteration in cellular energetic pathways. KPT-9274 given orally at 150 mg/kg 5 days/week for 4 weeks dramatically reduced PNET sub-cutaneous tumor growth. Residual tumor analysis demonstrated target engagement in vivo and recapitulated in vitro results. Our investigations demonstrate that PAK4 and NAMPT are two viable therapeutic targets in the difficult to treat PNET that warrant further clinical investigation.

## 1. Introduction

Although pancreatic neuroendocrine tumors (PNET) are rare neoplasia that represent <3% of all pancreatic cancers [1], their incidence has increased over the past two decades [2]. The principal treatment for localized PNET is surgical resection. However, there is no curative therapy for patients with advanced unresectable or metastatic disease and unfortunately, the dismal outcome of PNET highlights the urgent need for identification and development of novel molecularly targeted therapies.

The mechanistic target of rapamycin (mTOR) inhibitor everolimus is an FDA approved treatment for progressive, well-differentiated, locally advanced, or metastatic PNET [3]. Nevertheless, only a subset of the patients responds to everolimus [3]. Resistance to everolimus has been primarily attributed to the activation of protein kinase B (Akt) and phosphoinositide 3-kinase (PI3K), by means of mTOR complex 2 (mTORC2) and insulin growth factor (IGF) and receptor (IGFR) signaling [4]. Along these lines, PI3K and mTOR dual inhibition strategy has shown promising activity in preclinical models of PNET [5]. Additional pathways involving focal adhesion kinase (FAK) have also been identified to play a role in the development of resistance [6]. However, approaches targeting PI3K, Akt, mTORC2, IGF, or FAK have not made any meaningful clinical impact on metastatic PNET [7]. Such persistent failures in targeted approaches indicate that there is a void in our understanding of the mechanisms of therapy resistance in PNET. There is an urgent need for the identification of molecularly driven targets that can simultaneously block multiple resistance pathways to improve treatment outcomes.

Frequent mutations in multiple endocrine neoplasia 1 (MEN1; 44%), death domain-associated protein (DAXX)/chromatin remodeler (ATRX; 43%), mTOR (15%) pathway genes, and Von Hippel Lindau (VHL) alongside several other hereditary disorders are observed in PNET [8]. Loss of *VHL* has been linked to enhanced tumor aerobic glycolysis (Warburg effect) [9]. In this scenario, cancer cells rely more heavily on a nicotinamide adenine dinucleotide (NAD) pool that is a crucial co-factor in the redox reactions of metabolic pathways of cancer cells with high aerobic glycolysis [10]. This over-dependence on NAD may provide actionable therapeutic avenues within the NAD salvage pathway in PNET.

The mTOR pathway regulatory proteins belonging to the p21-activated kinase (PAK) family are crucial effectors of the Rho family of GTPases (RhoA, Rac1, and Cdc42) and act as regulatory switches that control important cellular processes including motility, proliferation, and survival [11]. When activated by mutation or overexpression, most PAK isoforms (Group I: PAK 1, 2, 3 or Group II: PAK 4, 5, 6) have oncogenic signaling effects. PAK4 is the main effector of cell division control protein 42 homolog (Cdc42); thus, it acts as a critical mediator of the Rho family of GTPases [12]. PAK4 protein by virtue of its ability to engage multiple ligands has been shown to regulate a repertoire of signaling pathways including PNET resistance drivers mTORC1, mTORC2, PI3K, mitogen-activated protein kinase 1 (ERK), FAK, RAPTOR independent companion of mTOR complex 2 (RICTOR), β-catenin, and IGF-1 [13,14]. Relevant to pancreatic cancer, in early studies, copy number alteration analysis showed amplification of PAK4 in pancreatic ductal adenocarcinoma (PDAC) patients [15]. Studies have also linked such amplification to cell migration, cell adhesion, and anchorage-independent growth [16]. Studies in non-PNET models have clearly demonstrated that PAK4 amplification can cause activation of Akt, ERK, mTORC1, mTORC2 [17], β-catenin, and IGF-1 [18]—the major players of drug resistance in PNET. Linking PAK signaling to NAD has shown that blocking Rho-kinase can ameliorate metabolic disorders through the activation of the AMP-activated protein kinase (AMPK) pathway in mouse models [19]. Our group has earlier shown that targeting PAK4 can suppress PDAC proliferation and stemness in vitro and in vivo [20]. At the same time, independent studies have verified that NAMPT inhibition could become a synthetic lethality in PDAC [21]. Collectively, these studies indicate that PAK4-NAMPT could also become potential therapeutic targets for therapy-resistant PNET.

In this report, we show for the first time that PNETs depend on the PAK4-NAMPT axis for their subsistence. We demonstrate that targeting of PAK4-NAMPT with the clinical stage dual inhibitor, KPT-9274, could be a viable therapeutic strategy for this incurable and deadly disease.

## 2. Results

### 2.1. PAK4 and NAMPT Are Overexpressed in PNET

**Figure 1:**
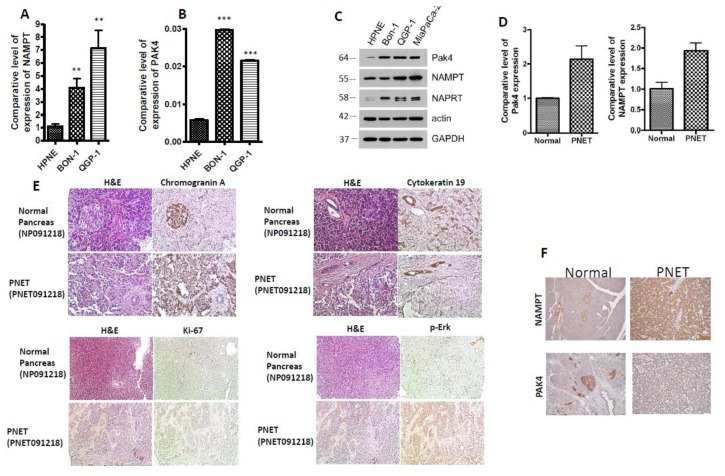
p21-activated kinase 4 (PAK4) and nicotinamide phosphoribosyltransferase (NAMPT) are significantly up-regulated in pancreatic neuroendocrine tumors (PNET) cell lines and patient primary tissue. (**A**,**B**) RT qPCR comparing the basal expression level of PAK4 and NAMPT in normal pancreatic cells (HPNE), and PNET cell lines BON-1 and QGP-1 (** *p* < 0.01; *** *p* < 0.005). Each expression level was normalized with actin mRNA. Graph representative of three independent experiments. (**C**) An equal amount of proteins were resolved on a 10% PAGE followed by Western blot comparing the basal expression level of PAK4, NAMPT, and (nicotinic acid phosphoribosyltransferase) NAPRT in normal HPNE, PNET cell lines BON-1 and QGP-1, and pancreatic ductal adenocarcinoma cell line Miapaca-2. β-actin was used as a loading control. (**D**) RT qPCR comparing the basal expression level of PAK4 and NAMPT in patient-derived tissue and matched normal tissue from the same patient ((*n* = 1) normalized with actin mRNA). (**E**) Immunohistochemistry (IHC) for chromogranin A, cytokeratin 19, Ki-6, and p-ERK in PNET and matched normal tissue from the same patient (200 × magnification) (**F**) IHC staining for NAMPT and PAK4 in well-differentiated low-grade PNETs (*n* = 15) showing (100 × magnification) positive staining in the PNET tissue (right panel) compared to normal controls (left panel).

To investigate the implication of PAK4 and NAMPT in PNET therapy resistance and survival, we first evaluated the basal expression level of these two proteins in PNET cell lines (BON-1 and QGP-1) and patient-derived tumor tissue using Western blotting and RT-qPCR. Compared to normal pancreatic cells (HPNE), the expression of NAMPT and PAK4 was found to be higher in the PNET cell lines BON-1 and QGP-1 (Figure 1A–C). It is important to note that BON-1 and QGP-1 are the only available cellular models to study PNET hitherto. PNET tissue and matched control from the same patient were examined via immunohistochemistry (IHC). The expression levels of PAK4 and NAMPT were found to be significantly higher in PNET patient tissue compared to normal matched tissue (*n* = 1) (Figure 1D). The PNET patient tissue expressed chromogranin A, but not cytokeratin 19, confirming the PNET (not PDAC) diagnosis of the donor patient (Figure 1E to right panel). Chromogranin A is a marker for neuroendocrine tissue while cytokeratin 19 is a marker for PDAC ([22,23] respectively). This was a low-grade tumor marked by very low Ki-67 expression (Figure 1E lower left panel). We also found an elevated expression level of p-ERK in the PNET tissue relative to the normal tissue (Figure 1E lower right panel). We further evaluated the expression of NAMPT in 15 well-differentiated PNET tissue using IHC. As can be seen from results of Figure 1F, compared to normal control tissue (staining score 0–1), the PNET tissue stained positively for NAMPT and PAK4 expression. In the NAMPT group, all the cases showed diffuse staining within tumor cells; the staining intensity was 1 in one case and 3 in 14 cases, respectively. In PAK4 group, among 15 cases, 13 showed diffuse staining within tumor cells; the staining intensity was 1 in six cases and 2 in nine cases, respectively. Both NAMPT and PAK4 were predominantly localized in the islet cells of normal tissue. However, in the tumors, staining was diffused throughout the tissue. The aberrant expressions of PAK4 and NAMPT in PNET cellular models and patient-derived tissue suggest there is a role for these proteins in maintaining PNET initiation or development that warrants further evaluation.

### 2.2. PAK4 and NAMPT Promote PNET Survival

**Figure 2:**
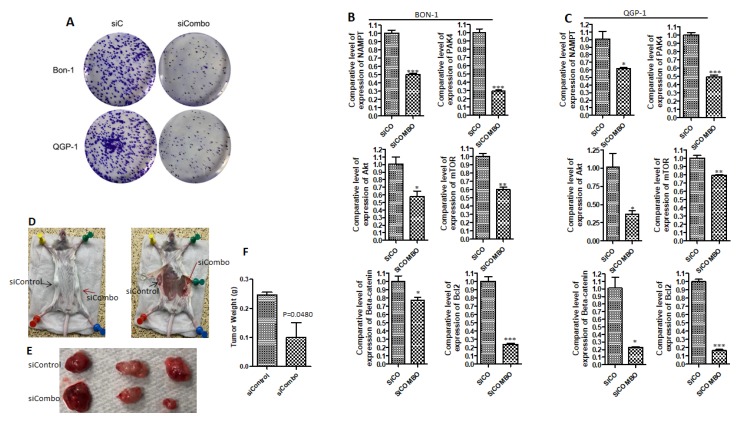
PAK4-NAMPT RNA interference inhibits the growth of PNET cellular model and suppresses survival factors. (**A**) Colony formation assay to show that PAK4-NAMPT RNA interference induces long term growth inhibition in PNET cell lines. (**B**,**C**) RT qPCR showing the down-regulation level of PAK4, NAMPT, survival factors, and Bcl2 after RNA interference in BON-1 and QGP-1 respectively (* *p* < 0.05; ** *p* < 0.01; *** *p* < 0.005). Each expression level was normalized with actin mRNA. (**D**) Animal images (*n* = 3) showing significant growth inhibition of siPAK4-siNAMPT silencing in BON-1 tumor. (**E**) Photographs of excised tumors showing a significant reduction in tumor size in two mice out of three. (**F**) Graphical representation of the combined tumor weight of the control and PAK4-NAMPT siRNA treatment showing statistically significant inhibition (*p* = 0.0480).

To demonstrate whether PAK4 and NAMPT promote PNET survival, we used an RNA interference (RNAi) strategy. PAK4-NAMPT RNAi suppressed the growth of BON-1 and QGP-1 cells in a statistically significant manner (*p* < 0.05 in colony formation assay) (Figure 2A). RT-PCR confirmed that PAK4-NAMPT RNAi downregulates the expression level of PAK4 and NAMPT in BON-1 and QGP-1 (Figure 2B,C, respectively). Next, we examined whether downregulation of PAK4 and NAMPT inhibits the expression of pro-survival factors associated with therapy resistance in PNET. As expected, PAK4-NAMPT RNAi caused a statistically significant reduction in the expression level of survival factors such as Akt, mTOR, and β-catenin in PNET cellular models (Figure 2B,C, respectively). Additionally, the anti-apoptotic player Bcl-2 was also significantly downregulated upon PAK4-NAMPT RNAi (Figure 2B,C, respectively). More importantly, PAK4-NAMPT RNAi inhibited the growth of BON-1 tumor in vivo (Figure 2D–F). Taken together, these results clearly show that PAK4 and NAMPT play a crucial role in the biology of PNET, which led us to investigate the impact of chemical inhibition using the PAK4-NAMPT dual inhibitor KPT-9274 and related analogs.

### 2.3. KPT-9274 and Analog KPT-7523 Decrease PNET Cell Survival and Growth

**Figure 3:**
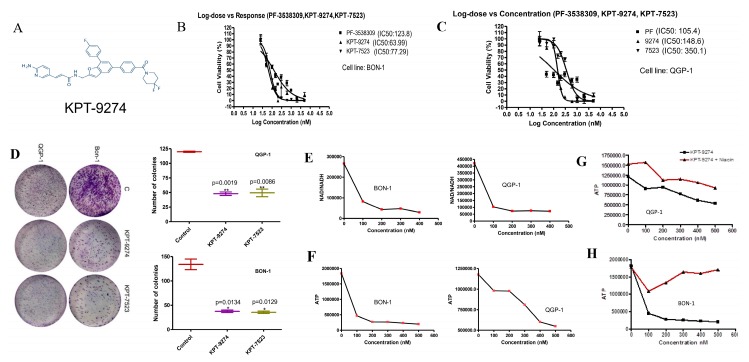
PAK4-NAMPT dual inhibitors effectively block cell proliferation and cause energy collapse in a PNET cellular model. (**A**–**C**) Graphs representing growth curves in the presence of different PAK-NAMPT dual inhibitors. IC_50s_ of KPT-9274, and analog KPT-7523 or +ve control PF in BON-1 and QGP-1 cell lines at 72 h using MTT assay was calculated using GraphPad Prism software. (**D**) (Left panel) Colony formation assay post-KPT-9274 and analog KPT-7523 treatment in BON-1 (at 2X IC_50_ for each cell line); (Right panel) colony quantification. (**E**) NAD Cell Titer-Glo assay was performed according to the manufacturer’s protocol. Graphs showing a reduction of NAD pool after KPT-9274 treatment in BON-1 and QGP-1. (**F**) ATP Cell Titer-Glo assay showing a reduction of ATP pool level after PAK4 and NAMPT inhibition. Graphs representative of two independent experiments. (**G**,**H**) PNET cell lines grown in KPT-9274 and niacin (1:1 ratio) followed by NAD Cell Titer-Glo assay as above.

To investigate the role of PAK4 and NAMPT signaling on the proliferation and survival of PNET cell lines, we treated BON-1 and QGP-1 with an escalating concentration of KPT-9274 (structure given in Figure 3A) and an analog KPT-7523 in the presence or absence of PAK4 and/or NAMPT specific inhibitors PF3758309 and FK866, respectively. Figure 3B,C shows the IC_50s_ of KPT-9274 and KPT-7523 in BON-1 and QGP-1 cell lines. BON-1 cells were relatively sensitive to treatment with KPT-9274 (IC_50_ 77.29 nM) and KPT-7523 (IC_50_ 63.99 nM). In the QGP-1 cell line, the IC_50s_ of KPT-9274 and KPT-7523 were found to be ~140.6 and 350.1 nM, respectively. Similar efficacy was seen with positive controls PF3758309 (Figure 3B,C). These results suggest that PNET cellular models are sensitive to dual inhibition of PAK4 and NAMPT. Earlier, we showed that KPT-9274 and analogs have limited activity against normal human pancreatic ductal epithelial (HPDE) cells. Inhibition of cell proliferation (MTT) assays were supported by a clonogenic assay where we observed a significant reduction in colony formation post-KPT-9274 or KPT-7523 treatment in the PNET cell lines (Figure 3D). In line with the NAMPT inhibitor mechanism of action, the treatment of PNET cell lines with KPT-9274 resulted in a significant reduction of cellular NAD (Figure 3E) and ATP levels (Figure 3F) in both BON-1 and QGP-1 respectively. Therefore, in order to demonstrate specificity of KPT-9274 towards NAMPT inhibition, we tested whether treatment with niacin in combination with KPT-9274 would rescue NAD biosynthesis in our PNET cellular models (by promoting the Preiss Handler pathway). We observed that a combination of KPT-9274 and niacin (1:1 ratio) rescued ATP pool level in BON-1 and QGP-1 (Figure 3G,H). Fortified by these results in our PNET cellular models, the combination of KPT-9274 with FDA approved therapeutic everolimus was further characterized.

### 2.4. KPT-9274 Synergizes with Everolimus in PNET Cellular Models

**Figure 4:**
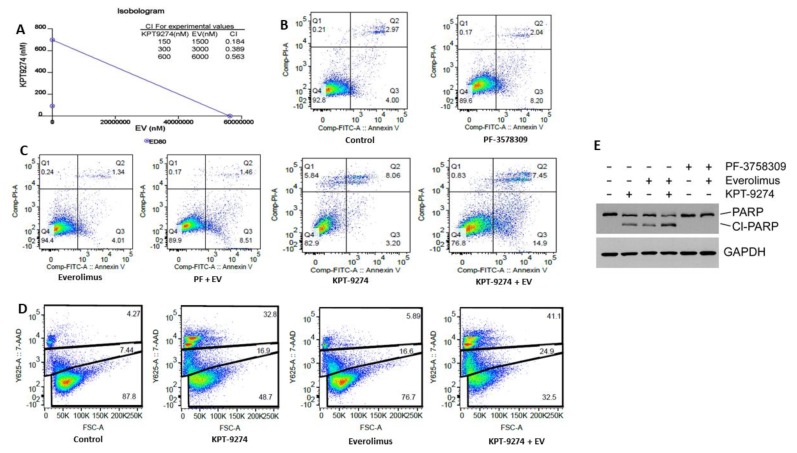
KPT-9274 synergizes with everolimus. QGP-1 cells were grown in 96-well plates and exposed to indicate concentrations of different drugs as described in Methods. (**A**) Isobologram showing the synergistic interaction of KPT-9274 with everolimus (EV) at 72 h MTT assay (QGP-1 cell line). (**B**,**C**) PNET cell lines were grown at a density of 30,000 cells per well in six-well plates in duplicate and exposed to different drugs 600 nM of KPT-9274; 6 μM everolimus or their combination for 72 h. At the end of the treatment period, cells were trypsinized and collected in pre-labeled tubes. The cell pellet was suspended in annexin V FITC binding buffer and apoptosis analysis was performed according to manufacturer provided protocol (Biovision Danvers MA). (**D**) A total of 30,000 cells were grown in six-well plates in duplicate and exposed to the same drug concentration. At the end of the treatment period, cells were stained with 7AAD as described in Methods. After staining, analysis for dead cells was performed using flow cytometry. (**E**) QGP-1 cells were grown in 100 mm petri plates overnight. The next day the cells were exposed to either KPT-9274 (600 nM); everolimus (6 μM) or their combination for 72 h. PF-3758309 was used as a positive control at 125 nM for 72 h with everolimus at (6 μM).

The two PNET cell lines used in this study are inherently resistant to everolimus (BON1 IC_50_ ~32.42 μM and QGP-1 IC_50_ ~27.63 μM; Figure S1). Their inherent resistance to the mTOR inhibitor prompted us to investigate whether PAK4-NAMPT dual inhibition can sensitize the PNET cell lines to everolimus. In the MTT assay, KPT-9274 clearly demonstrated synergy with everolimus. More striking was the observation that a synergistic combination index less than 1 (CI < 1) was observed for all doses tested (Figure 4A). Supporting evidence for this synergy came from annexin V FITC and 7AAD apoptosis analysis. KPT-9274 as a single agent demonstrated minimal apoptosis (Figure 4B,C). However, the combination of KPT-9274 with everolimus showed a statistically significant enhancement in apoptosis compared to single-agent treatment (*p* < 0.01). Similar results were found in using another assay (7AAD) where significantly enhanced apoptosis was observed in the combination with everolimus (Figure 4D). These results were further supported by Western blot analysis. We observed superior PARP cleavage in KPT-9274-everolimus compared to single-agent treatment (Figure 4E). In addition, we tested the combination of KPT-9274 with the next generation mTOR inhibitor INK-128. As anticipated, the combination demonstrated marked enhancement in apoptosis compared to single-agent treatment (Figure S1). We further evaluated several other standard of care treatment combinations. We also tested several other combinations including KPT-9274-sunitinib (receptor tyrosine kinase inhibitor) and KPT-9274-FAKi. Synergy was only observed with sunitinib (Figure S2). More importantly, we observed significant decrease in phosphorylation of Akt in response to KPT-9274-everolimus (Figure S4C). These studies strengthen the proposed hypothesis that the combination of KPT-9274 with everolimus could become a feasible strategy for resistant PNET that needs further characterization.

### 2.5. Molecular Analysis of KPT-9274-Everolimus Combination

**Figure 5:**
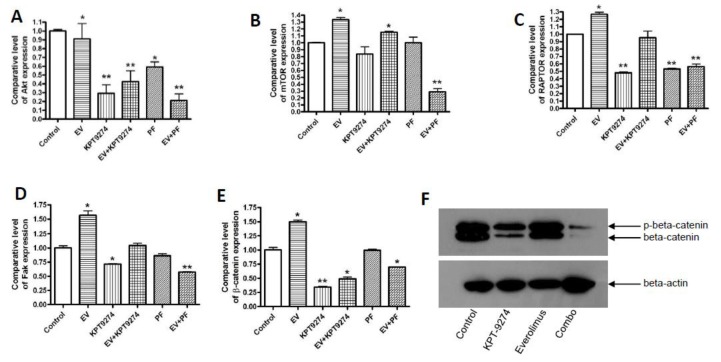
Molecular analysis of the KPT-9274-everolimus combination. (**A**–**E**) RT qPCR in QGP-1 cells showing the down-regulation of survival factors after KPT-9274 treatment. Cells were seeded at a density of 50,000 cells per well in six-well plates in duplicate and exposed to KPT-9274 (600 nM); PF-3578309 (300 nM); everolimus (6 μM) or their combination for 72 h. After each treatment period, pooled RNA from each group was isolated and subjected to real-time qPCR. The expression level of Akt, mTOR, RAPTOR, β-catenin, and focal adhesion kinase (FAK) mRNA was normalized with GAPDH mRNA. * *p* < 0.05; ** *p* < 0.01. (**F**) Western blotting analysis for β-catenin expression changes in QGP-1 cells exposed to KPT-9274 (600 nM); everolimus (6 μM) and their combination for 72 h.

As anticipated, the KPT-9274-everolimus combination or the PF-03798309-everolimus combination showed statistically significant down-regulation of a number of well-recognized PNET resistance molecules including mTOR, Akt, the regulatory-associated protein of mTOR (RAPTOR), FAK, and β-catenin in QGP-1 cell lines (Figure 5A–E RT-PCR ** *p* < 0.01). The results also show that KPT-9274 treatment causes a down-regulation of mutant *MEN1* downstream of the Wnt/β-catenin signaling in QGP-1 cells (Figure 5F). Further supporting these findings, work done by an independent group in a kidney cancer model showed that KPT-9274 caused complete inhibition of β-catenin in vitro and also in tumor tissues post oral drug treatment [24].

### 2.6. Metabolomic Analysis of PNET Cells Treated with KPT-9274

**Figure 6:**
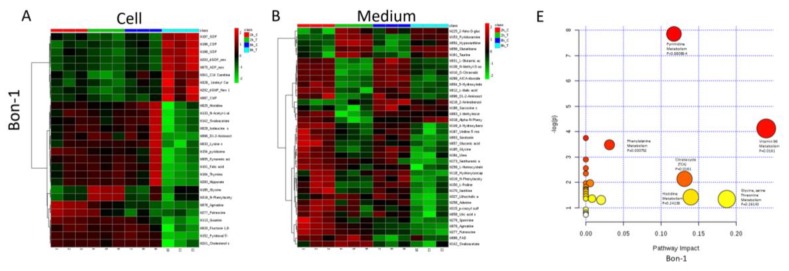

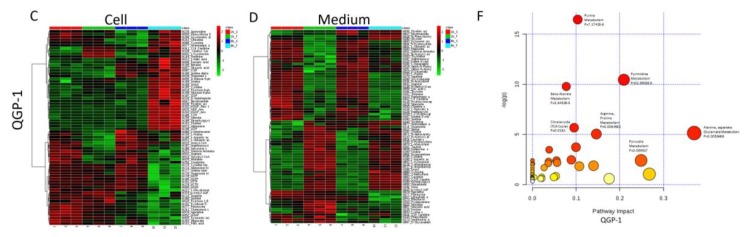
Metabolomic analysis of metabolites altered upon KPT-9274 treatment. First, 2 × 10^6^ QGP-1 or Bon-1 cell lines were seeded in a 60 × 15 mm petri dish in appropriate medium supplied with 10% FBS and 1% Pen Strep for each cell line, and allowed to grow overnight at 37 °C in a 5% CO_2_ incubator until the cells were 60% to 80% confluent. The next day cells were exposed to 600 nM of KPT-9274 for 2 and 8 h. Each cell line and treatment duration were performed in triplicate. After the treatment period, the supernatant from each condition was collected in a labeled 1.5 mL tube and the cells were washed two times with ice-cold PBS. After carefully removing PBS, the cells were collected using 1 mL of cold methanol. Changes in metabolites were detected using liquid chromatograph-mass spectrometry. Heat map of Bon-1 cells exposed to KPT-9274 for 2 and 8 h followed by the analysis of changes in metabolites in the cell (**A**) and medium (**B**). Heat map of QGP-1 cells exposed to KPT-9274 for 2 and 8 h followed by the analysis of changes in metabolites in cells (**C**) and medium (**D**). Metabolic pathway impact analysis of significantly upregulated metabolites by MetaboAnalyst 4.0 in Bon-1 (**E**) and QGP-1 (**F**).

We next performed a metabolomic analysis to examine metabolites modulated by PAK4-NAMPT dual inhibition in PNET cellular models. Exposure of QGP-1 or BON-1 cells to KPT-9274 for 2 and 8 h resulted in statistically significant alterations in a series of metabolites related to the cellular energetics in both cell and extracellular culture media (Table 1 and Table 2; heat map in Figure 6A–D; and Figure S3A–F). The impact on metabolites was greater at 8 h in both cell and media samples compared to 2 h. Pathway analysis showed that the majority of the molecules altered by KPT-9274 treatment were part of the pyrimidine biosynthesis pathway (pyrimidine biosynthesis pathway *p* = 3.8808 × 10^−4^ for Bon-1 and *p* = 2.8901 × 10^−5^ for QGP-1; Figure 6E,F). These results show that KPT-9274 induces a metabolic change in the PNET cell lines and further supports the use of metabolism targeted agents against this therapy-resistant tumor.

### 2.7. KPT-9274 Shows Single Agent Anti-Tumor Activity in PNET

**Figure 7:**
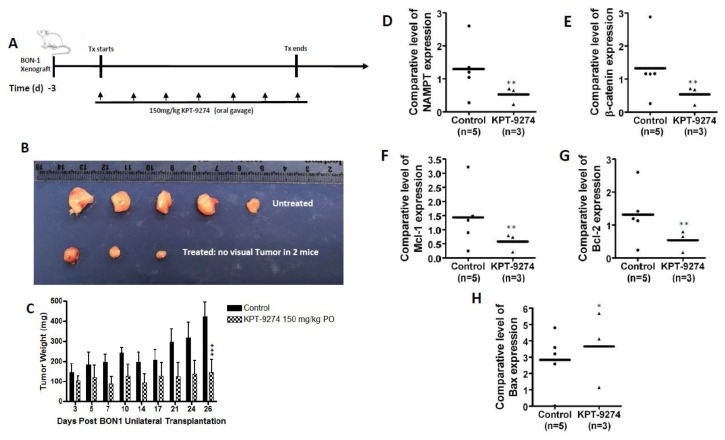
KPT-9274 inhibits BON-1 growth in vivo. BON-1 cells were grown as a subcutaneous xenograft in ICR-SCID mice (see methods). (**A**) Graphical representation of the treatment scheme of the in vivo study. (**B**) Gross visualization of excised tumors (five mice in total were used). Two mice showed no visual tumors. (**C**) Graphical representation of tumor weight during the treatment at the indicated days (*** representing *p* = 0.008 for combined tumor weight of treated group compared to control group at the end of the treatment). (**D**–**H**) RT qPCR in BON-1 residual tumor cells showing down-regulation of NAMPT, β-catenin, Bcl2, and Mcl1 and activation of Bax. * representing *p* < 0.05; ** representing *p* < 0.01.

Earlier studies from our group and others have demonstrated remarkable anti-tumor activity of KPT-9274 in solid (breast, colon, and kidney) and hematologic (NHL, CLL, and MM) tumor xenograft models [25,26,27,28]. Our group has also demonstrated the activity of KPT-9274 in PDAC xenograft in vivo [29]. Driven by these encouraging animal studies, we evaluated the anti-tumor activity of KPT-9274 in BON-1 xenograft. KPT-9274 treatment at 150 mg/kg twice a day, five days a week for 3–4 weeks (see schema in Figure 7A) resulted in a statistically significant reduction of tumor growth of BON-1 xenograft (Figure 7B (gross tumor images), and Figure 7C (tumor weight) *p* < 0.01). Molecular analysis performed on residual tumors shows the PAK4-NAMPT dual inhibitor suppresses the expression levels of NAMPT and the downstream target of PAK4; β-catenin (Figure 7D,E). Analysis of anti-apoptotic markers was also in line with in vitro results; KPT-9274 suppresses the expression level of Mcl-1 and Bcl-2 (Figure 7F,G) with simultaneous activation of pro-apoptotic Bax (Figure 7H). Collectively these findings suggest that the PNET cell line (Bon-1) is sensitive to PAK4 and NAMPT dual inhibition.

## 3. Discussion

This study is the first to report the critical role of the PAK-NAMPT axis in PNET subsistence. We demonstrate that dual inhibition of PAK4 and NAMPT can block PNET proliferation in vitro and arrest tumor growth in mouse models. We also show that the PAK4-NAMPT dual inhibition can sensitize PNET cells to everolimus in vitro. These results put forth a novel therapeutic strategy for the difficult to treat subtype of pancreatic cancer.

Current understanding of the genetics of PNET points to the hyperactivation of genes in the mTOR pathway. This led to the FDA approval of the mTOR inhibitor everolimus for the treatment of PNET [30]. Although the median progression-free survival rate is 11 months for everolimus compared to 4.6 months with the placebo, most of the patients develop drug resistance indicating there is a void in our understanding of the mechanisms of resistance to mTOR inhibition in PNET. Resistance to everolimus has been linked to the activation of PI3K and Akt, by means of mTORC2 and IGFR signaling. Despite this knowledge, individual or combination approaches to target PI3K, Akt, mTORC2, or IGF have not yet made any meaningful impact on advanced unresectable or metastatic PNET in the clinic. Such recurrent failure in targeted approaches suggests that we do not fully understand the mechanisms of resistance in PNET.

Aberrant expression of PAK4 and NAMPT is driven by a variety of genetic changes play a critical role in the survival of several cancers. These two proteins have been shown to regulate a myriad of pathways associated with therapy resistance in PNET [31]. Previous studies hypothesize that under hypoxic conditions, PAK4 interacts with the Rho family GTPases to regulate the actin cytoskeleton and control AKT-mTOR-4E-BP1 signaling in cancers [32]. In addition, NAMPT, the rate-limiting enzyme in the salvage pathway of NAD biosynthesis in mammals, has been implicated in the regulation of mTOR signaling [33]. Schuster and colleagues have shown that inhibition of NAMPT results in the activation of AMPK and consequent suppression of mTOR [34]. More importantly, in a recent study, Chainnaiyan and colleagues evaluated more than 200 patients’ samples of gastroenteropancreatic neuroendocrine tumors and showed that NAMPT is one of the mechanistic dependencies of neuroendocrine tumors [35]. These multiple lines of evidence point to a critical role of PAK4-NAMPT axis in PNET survival making them attractive therapeutic targets for this disease.

The synthetic lethality of our approach lies in the way cancer cells generate NAD. There are three different pathways for NAD biosynthesis in mammals. The Preiss–Handler pathway is initiated with niacin (also known as nicotinic acid or vitamin B3) as a substrate and is catalyzed by the nicotinic acid phosphoribosyltransferase (NAPRT1) [36]. The de novo pathway is initiated with tryptophan and includes nine steps; thus, more energy is required for this pathway. Most cancer cells do not rely on this pathway for NAD biosynthesis [37]. The salvage pathway starts with nicotinamide (another form of vitamin B3) and is catalyzed by NAMPT [38]. Although NAPRT1 remains functional in normal tissue, this critical enzyme has been shown to be silenced in tumors via promoter hypermethylation [39]. Indeed, studies have shown that normal cells supplied with niacin can continue synthesizing NAD through the Preiss–Handler pathway despite NAMPT inhibition. Supporting this, our results show that the addition of niacin reverses the ATP collapse in the PNET cell line to some extent. This in principle can provide a therapeutic window for cancer cell selective inhibition of NAMPT and resultant metabolic collapse of tumor cells.

Our results show that PAK4 and NAMPT inhibition synergizes with everolimus. A caveat to these studies is that the doses used for everolimus are significantly higher and may not be pharmacologically relevant. To address this, we are investigating the preclinical efficacy of everolimus in combination with KPT-9274 in xenograft derived from QGP-1 and BON-1. However, such studies are beyond the scope of this manuscript. It is important to note that lack of a sizable number of validated PNET cell lines is a significant problem that impacts research in this intractable disease. The studies presented here do have some limitations given that the activity of PAK4-NAMPT dual inhibitor was shown in only two PNET cell lines. Unfortunately, lack of good PNET cell line models restricts our ability to test our hypothesis in a larger number of cells. The use of patient-derived tumor models can certainly circumvent this issue and such studies are forthcoming. Collectively, our findings indicate that PNET tumors can be screened for NAPRT silencing to improve the therapeutic index when co-dosed with niacin.

## 4. Materials and Methods

### 4.1. Cell Lines and Reagents

PNET cellular models are limited to BON-1 and QGP-1. These two cell lines are the only available PNET cells used in research. QGP-1 cells were purchased from JCRB cell bank (Osaka, Japan). BON-1 cells were obtained under a material transfer agreement from Dr. Hellmich and Dr. Townsend (University of Texas Medical Branch, Galveston, TX, USA). Dr. Michel M. Ouellette (Department of internal medicine, Division of Gastroenterology and Hepatology, University of Nebraska Medical Center, Omaha, NE, USA) donated human pancreatic nestin-expressing (HPNE) cells. HPNE cells were authenticated using STR profiling. All other cells were not authenticated. All cells were maintained at 37 °C and 5% CO_2_. QGP-1 cells were grown in RPMI (Gibco) culture medium and BON-1 cells were maintained in DMEM/F12 Ham (Thermo Fisher, Waltham, MA, USA) culture medium. Each culture medium was supplemented with 10% FBS (Atlanta Biologicals, Atlanta, GA, USA) and 1% penicillin/streptomycin (Gibco by Life Technologies). PNET tissue blocks were collected under an approved IRB protocol (Emory University, Atlanta, GA, USA). The PAK4-NAMPT dual inhibitors (KPT-9274 and analog KPT-7523) were obtained from Karyopharm Therapeutics (Newton, MA, USA). KPT-9274 is a CRISPRres validated NAMPT inhibitor with dual inhibitory activity of PAK4 [40]. The following drugs were purchased from Selleckchem (Houston, TX, USA): Everolimus ((RAD001) mTOR inhibitor), INK-128, FK866 (APO866/Daporinad specific inhibitor of NAMPT), and PF3758309 (specific inhibitor of PAK4). All drugs were dissolved in dimethyl sulfoxide (DMSO). siPAK4 and siNAMPT were purchased from Santa Cruz Biotechnology (Dallas, TX, USA). Primary and secondary antibodies were purchased from multiple vendors including Cell Signaling Technology, Proteintech, and/or Santa Cruz Biotechnology.

### 4.2. Small Interference RNA and Transfection

To elucidate the role of PAK4 and NAMPT in PNET survival and drug resistance, we used siRNA-silencing technology. In a 60 mm petri dish, 50,000 BON-1 cells and 100,000 QGP-1 cells were seeded using 2 mL of antibiotic-free normal growth medium. The cells were then incubated at 37 °C in a CO_2_ incubator until 60–80% confluence. The next day, BON-1 and QGP-1 cells were transfected for 8 h using a mixture of each specific siRNA (siPAK4 + siNAMPT) at a concentration of 1 μg. After 8 h treatment, the cells were washed with PBS and placed in the incubator for 48 h. The cells were then collected, seeded again, and transfected one more time (double transfection). After the second siRNA treatment period, the cells were washed with PBS and 1000 cells were seeded in petri dishes and incubated for 3–4 weeks. With the remaining population of cells, siRNA knockdown efficiency was analyzed using RT-qPCR.

### 4.3. MTT Assay

PNET cell lines were grown to a density of 3000–5000 cells in 96-well plates overnight. The next day the cells were exposed to increasing concentration of either KPT-9274, KPT-7523, PF3758309, or FK866 in the presence or absence of equimolar concentrations of everolimus or INK128 (to satisfy isobologram synergy analysis) for 72 h. At the end of the treatment, 20 μL of MTT reagent was added in each well and further incubated for 2 h. Following this, 100 μL of DMSO was added to each well and the plates were incubated in dark on a high-speed shaker for 30 min. The formazan developed was read using a plate reader at 570 nm. The raw data (six replicates per treatment condition) was plotted as a bar graph or growth curves using GraphPad Prism software. For combination studies, the values were subjected to isobologram analysis using CalcuSyn Software.

### 4.4. Colonogenic Assay

First, 50,000 BON-1 cells and 100,000 QGP-1 cells were seeded in 60-mm petri dishes and incubated for 24 h until the cells were 80% confluent. The next day, cells were treated with escalating concentrations of KPT-9274 or analog KPT-7523 for 72 h. FK866 and PF3758309 were used as positive controls. After the treatment period, cells were washed with warm PBS and 1000 cells were collected from each treatment condition and re-seeded in 15 × 60 mm petri dishes and allowed to grow for 4 weeks (BON-1) and 6 weeks (QGP-1) at 37 °C in a 5% CO_2_ incubator. After the incubation period, the supernatant was removed from each dish, and cells were exposed to 2 mL of methanol for 5 min. Next, colonies were stained with 2% crystal violet, allowed to dry, photographed, and quantified.

### 4.5. Annexin V and 7AAD Apoptosis Analysis

Apoptotic cells were sorted using Annexin V FITC (Biovision, Danvers, MA, USA) and 7-amino-actinomycin D (Invitrogen Life Technology, Carlsbad, CA, USA) according to the manufacturers’ protocol (Biovision, Danvers, MA, USA). PNET cell lines were seeded in 60-mm petri dishes. The next day cells were treated with single-agent KPT-9274, analogs, everolimus, INK128, or their combination at the indicated concentrations and incubated for 72 h. After the treatment period, cells were washed with PBS, trypsinized and stained with Annexin V and Propidium Iodide (Annexin V FITC) [41] or 7-AAD viability staining solution (7-AAD) [42]. Stained cells were sorted using the Becton Dickinson flow cytometer at the Karmanos Cancer Institute Flow Cytometry Core.

### 4.6. Immunohistochemistry Analysis

Under an IRB approved protocol, we were able to obtain a pancreatic neuroendocrine tumor tissue with matched control from the same patient. The date when the tumor was obtained was 09/12/2018 and therefore we designate this with the date. We took a portion of normal and tumor tissue for IHC and RT-PCR analysis. The remaining tissue was implanted in mice to develop the Patient derived tumor. We obtained patients’ consent for all primary tissues used in this study. All specimens were fixed in 10% formalin, embedded in paraffin, and cut into 4-μm thick slides. The slides were dewaxed and stained with hematoxylin and eosin (H and E). Then H and E slides were reviewed to confirm that cancer cells were present. Next, the immunohistochemically stained slides (PAK4 and NAMPT) were evaluated with appropriate positive and negative controls, based on the staining intensity and percentage of tumor cells with staining. The staining intensity was graded as 0 (no staining), 1 (weak staining), 2 (medium staining), or 3 (strong staining as positive control). The percentage of tumor cells with staining was categorized as diffuse (>50%) or focal (<50%) staining. In PAK4 group, among 15 cases, 13 showed diffuse staining within tumor cells; the staining intensity was 1 in six cases and 2 in nine cases, respectively. In the NAMPT group, all the cases showed diffuse staining within tumor cells; the staining intensity was 1 in one case and 3 in 14 cases, respectively.

### 4.7. Western Blot Analysis

The QGP-1 cell line is known for its slow growth potential (doubling time (DT) = 3.5 days) while BON-1 cell line has a relatively fast growth potential (DT = 1.5 days). Therefore, 50,000 BON-1 cells or 100,000 QGP-1 were grown in 100-mm petri dishes overnight. The following day, each cell line was treated with specified concentrations of KPT-9274, KPT-7523, PF-3758308, Everolimus, FK866, and combination for 72 h. Then, 30 μg of protein extracts from cells (treated and vehicle) were separated in a 10% SDS-PAGE and transferred into a nitrocellulose membrane (GE Healthcare Life Sciences). Mouse monoclonal antibodies anti-PAK4 (CAT: sc-393367), anti-NAMPT (CAT: sc-393510), anti-NAPRT (CAT: sc-398404), anti-GAPDH (CAT: sc-365062) from Santa Cruz Biotechnology; anti-β-catenin (CAT: 9562S), anti-PARP (CAT: 9542S), anti-phospho-Akt (CAT:9271S), anti-Akt (from Cell Signaling Technology (Danvers, MA, USA) were used at a 1:1000 dilution in 3% non-fat milk PBS Tween-20. (anti-GAPDH and anti-β-actin (from Sigma CAT: A2228) (were used at 0.1:1000 dilution).

### 4.8. Quantitative Real-Time PCR

After the indicated treatments, RNA was isolated from treated cells and remnant BON-1 tumors. RT-qPCR was done using SYBR Green PCR master mix (Applied Biosystems, Foster City, CA USA) on a StepOnePlus Real-Time PCR System according to the manufacturer’s instructions. Multiple primers were used in this study: Akt (Forward: TTGTGAAGGAGGGTTGGCTG, Reverse: CTCACGTTGGTCCACATCCT). mTOR (Forward: TTCCGACCTTCTGCCTTCAC, Reverse: CCACAGAAAGTAGCCCCAGG). β-catenin (Forward: CGCCATTTTAAGCCTCTCGG, Reverse: CTCCTCAGACCTTCCTCCGT). RAPTOR (Forward: GACCTCGTGAAGGACAACGG, Reverse: CTTCCTGCCCCGTGTGATAG). Rictor (Forward: GGTGTTGTGACTGAAACCCG, Reverse: GTCATTCCGCCCTCGTACTC). Bcl2 (Forward: TGAACTGGGGGAGGATTGTG, Reverse: CGTACAGTTCCACAAAGGCA). Mcl1 (Forward: GCGGTAATCGGACTCAACCT, Reverse: CTCCCCTCCCCCTATCTCTC).

FAK (Forward: GGCTCCCTTGCATCTTCCAG, Reverse: AGTTGGGGTCAAGGTAAGCAG). Each sample was run in triplicates. The protocol for this PCR included a denaturation (95 °C for 10 min), then 40 cycles of amplification and quantification (95 °C for 15 s, 60 °C for 1 min).

### 4.9. Metabolomic Analysis.

In a 60 × 15 mm petri dish, we seeded 2 × 10^6^ BON-1 and QGP-1 cell lines and incubated the cells overnight at 37 °C in a 5% CO_2_ incubator until the cells were 80% confluent. Appropriate medium supplied with 10% FBS and 1% Pen Strep for each cell line was used. The next day, the cells were exposed to 600 nM of KPT-9274 (PAK4-NAMPT dual inhibitor) for 2 and 8 h. Each cell line and treatment duration were performed in triplicate. After the treatment period, the supernatant was collected from each condition in a labeled 1.5 mL eppendorf tube and cells were washed twice with ice-cold PBS. After washing the PBS was completely removed and cells were collected in 1 mL ice-cold methanol. Changes in metabolites were detected using liquid chromatograph-mass spectrometry at the Karmanos Cancer Institute Pharmacology Core.

### 4.10. Animal Studies

All studies were conducted under Wayne State University’s Institutional Animal Care and Use Committee approved protocol (18-12-0887). After adaptation in our animal housing facility, four 6-week-old female ICR-SCID mice (Taconic farms, New York, NY, USA) were subcutaneously injected with BON-1 cell lines. Cell suspension mixed with PBS (1 × 10^6^ in 200 μL) was loaded in BD 26G × 5/8 1 mL Sub-Q syringe and injected into the flanks of the donor mice. When tumor burden reached about 5–10% of the donor mice body weight (using a caliper and calculation to confirm the seize (L × W^2^/2)), the donor mice were euthanized, tumor harvested, and fragment implanted into recipient mice (*n* = 10). Three days post the implant, the recipient mice (*n* = 5) were treated by oral gavage with vehicle or diluent KPT-9274 ((*n* = 5) twice a day for 4 weeks). Tumor size and body weight were recorded 2–3 times weekly. In vivo siRNA: BON-1 cells were exposed to PAK4-NAMPT dual RNAi as described above. An equal number of control siRNA or PAK4-NAMPT siRNA cells were implanted subcutaneously in the flank of female ICR SCID mice (*n* = 3). After 8 weeks the tumors were harvested and weighed and photographed.

### 4.11. Statistical Consideration

Statistical evaluations were performed using GraphPad Prism 4 software. As needed, the data were subjected to an unpaired two-tailed Student’s *t*-test and two-way ANOVA and presented as mean ± standard error of the mean of at least three replicate experiments. A *p*-value < 0.05 was considered statistically significant. For the metabolomic statistical analysis, below level of quantification and 0 values with half of the lowest value that was greater than 0 were imputed to allow comparison between the targets. Due to the skewness of the data, the resultant measurement was log transformed. A generalized least squares model was run (estimating a different variance for each treatment group) comparing the log value between treatment groups for all comparisons.

## 5. Conclusions

PAK4 and NAMPT have remained non-druggable targets for many years. The first specific PAK4 inhibitor PF-3578309 was discontinued from a Phase I clinical trial (NCT00932126) study due to lack of objective response. This agent was a type I PAK4 kinase competitive inhibitor and a substrate for the multi-drug transporter (PGP) which was reflected in its poor pharmacokinetic characteristics. Similarly, FK866 the first NAMPT specific inhibitor was also discontinued from phase I/II (NCT00435084) studies due to lack of objective response. KPT-9274 is a type II PAK4 allosteric modulator that has been validated through CRISPRres (a CRISPR-Cas-based genetic screening approach) to specifically inhibit NAMPT. KPT-9274 remains the only drug in its class to be in Phase I clinical studies for the treatment of patients with advanced solid malignancies or non-Hodgkin’s lymphoma (NCT02702492). More significantly, these trials have incorporated pre-screening for NAPRT methylation and niacin-KPT-9274 as an arm to study rescue and the ability to enhance the dosing of the drug. In conclusion, our study illustrates the therapeutic potential of PAK4-NAMPT dual inhibition as a feasible strategy for the difficult to treat pancreatic neuroendocrine tumors.

## Figures and Tables

**Table 1:** List of metabolites altered after KPT-9274 treatment in the Bon-1 cell line. Each table shows the effect size (in log scale) and the *p*-value for the comparison of treatment. A *p*-value of less than 0.05 was considered significant at 5% level. A positive effect means that the target is higher in the treated group relative to the control group. A negative effect is the converse.

Metabolites	Treatment Effect	*p*-Value
Kynurenic acid	−2.173	0.026
Fructose 1,6-bisphosphate	−1.903	0.016
Histidine	−2.356	0.023
Phenylalanine	−1.076	0.044
Arginine	−1.693	0.025
Lysine (s)	−2.729	0.021
dUMP	−1.043	0.025
1-Methylhistidine	−1.381	0.045
2-Ketohexanoic acid	1.938	0.001
N-Alpha-acetyllysine	−1.866	0.047
ADP_new	1.410	0.001
CMP	1.610	0.023
DL-2-Aminooctanoic acid	−2.158	0.008
Folic acid	−2.659	0.007
Guanine	−1.380	0.010
N-Acetylornithine	−2.706	0.046
Phenyllactic acid	−1.514	0.047
Pyridoxal 5-phosphate	−2.006	6.05 × 10^−7^
pyridoxine	−1.682	0.046
Thymine	−1.959	0.027
Sarcosine (s)	−1.121	0.018
CDP	2.040	0.003
GDP	1.496	0.002
UDP	1.875	0.001
dGMP_New 1	1.465	0.015
dGDP_new	1.452	0.001
Cholesterol sulfate	−1.412	0.010
Hippurate	−1.911	0.027
p-cresyl sulfate	−1.536	0.049
Linoleyl Carnitine	1.224	0.033
C14 Carnitine (Myristoyl-L-carnitine)	1.307	0.040

**Table 2:** List of metabolites altered after KPT-9274 treatment in the QGP cell line. Each table shows the effect size (in log scale) and the *p*-value for the comparison of treatment. A *p*-value of less than 0.05 was considered significant at 5% level. A positive effect means that the target is higher in the treated group relative to the control group. A negative effect is the converse.

Metabolites	Treatment Effect	*p*-Value
Succinyl-CoA	−1.299	8.87 × 10^−4^
Fructose 6-phosphate	2.483	0.008
Arginine	−1.179	0.008
Picolinic acid	1.633	0.034
Nicotinamide	1.695	0.033
D-Ribose 5-phosphate	2.741	6.27 × 10^−6^
Adenosine	3.164	2.51 × 10^−6^
ADP_new	1.805	5.26 × 10^−8^
Aminoadipic acid	1.352	0.026
Cytosine	1.806	0.007
Glucose 6-phosphate	2.506	0.008
GMP_New	2.775	2.08 × 10^−4^
Ornithine	1.342	0.025
Uracil	3.249	0.001
Uridine	3.731	0.001
Adenosine triphosphate	−1.486	0.004
Cytidine triphosphate	−1.884	0.003
Guanosine triphosphate	−1.819	0.006
Uridine triphosphate	−1.394	0.008
dATP	−1.520	0.001
dCTP	−1.011	0.008
dGTP	−1.389	0.008
dTTP	−1.052	0.002
Argininosuccinic acid	−1.162	0.001
Sarcosine (s)	−1.266	0.010
Dimethylglycine+M191	1.571	0.001
Choline	1.509	0.001
Cytidine	2.923	0.001
CDP	2.720	2.06 × 10^−6^
GDP	1.622	0.004
UDP	2.185	0.001
dGMP_New 1	3.419	3.40 × 10^−8^
dGDP_new	1.811	2.91 × 10^−6^
D-Sedoheptulose 7-phosphate	1.285	0.008
N-Acetyl-glucosamine 1-phosphate	1.210	0.008
Citicoline	3.320	0.005
Adenine	1.726	0.001
Spermidine	2.009	0.013
C16:0 Carnitine (Palmitoyl-L carnitine)	3.023	0.013

## Supplementary Materials

The following are available online at https://www.mdpi.com/2072-6694/11/12/1902/s1, Figure S1: PNET cells are inherently resistant to mTOR inhibitors, Figure S2: KPT-9274 synergy analysis, Figure S3: Targeting PAk4-NAMPT Axis alters the metabolism of PNET cells, Figure S4A,B single siPAK4 and single siNAMPT in PNET cells, Figure S4C decrease in the expression of phospho-Akt after single agent KPT-9274 and combination with everolimus.
