# Supplementary material for: PAK4-NAMPT Dual Inhibition as a Novel Strategy for Therapy Resistant Pancreatic Neuroendocrine Tumors
Source: Cancers (Basel). 2019 Nov 29;11(12):1902. doi: 10.3390/cancers11121902 (PMC6966587; doi:10.3390/cancers11121902)
Supplement: Supplementary file 1 [file cancers-11-01902-s001.pdf]

cancers

Supplementary Materials

Figure 1

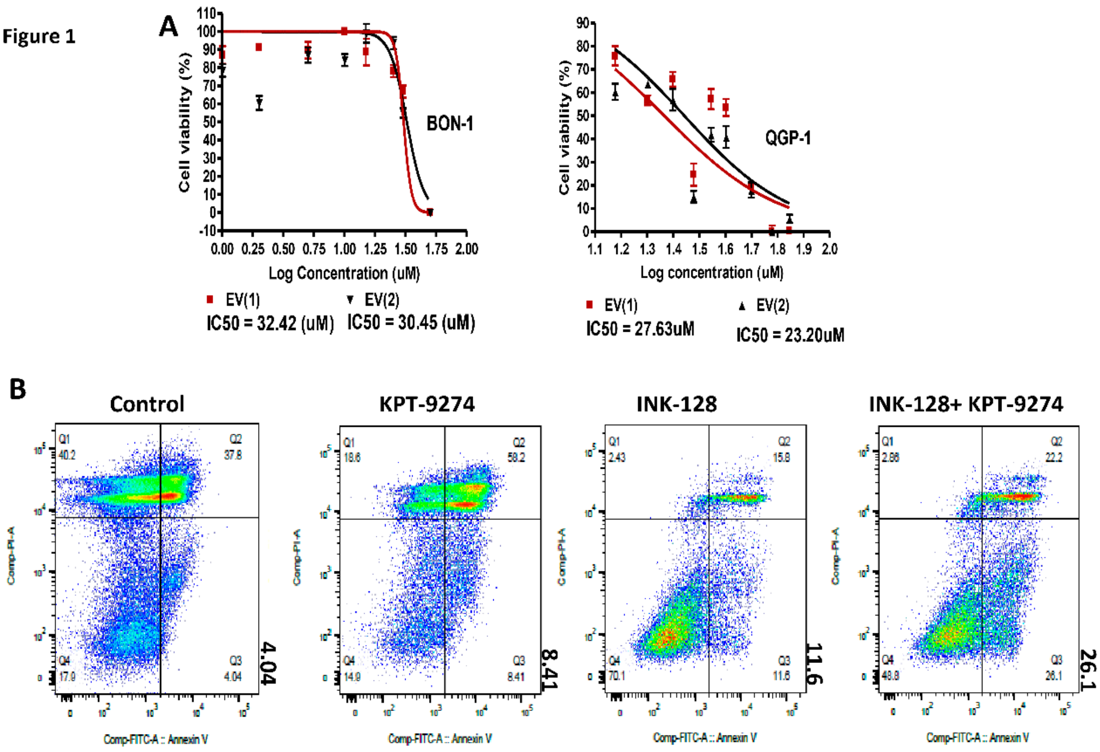

Figure S1. (**A**) PNET cells are inherently resistant to mTOR inhibitors. Bon-1 and QGP-1 cells were growth in 96 well plates overnight and exposed to increasing concentrations of everolimus (EV) for 72 h, (EV1 and EV2 represent two biological replicates). At the end of the treatment period, standard MTT assay was performed. Growth curves were developed using GraphPad Prism software. (**B**) KPT-9274 synergize with mTOR inhibitor INK-128. QGP-1 cells were grown at a density of 30,000 cell/well in six well plate overnight followed by incubation with either KPT-9274 (600 nM) or INK-128 (6 μM) or their combination for 72 h followed by Apoptosis analysis using standard protocol (Biovisions Danver MA).

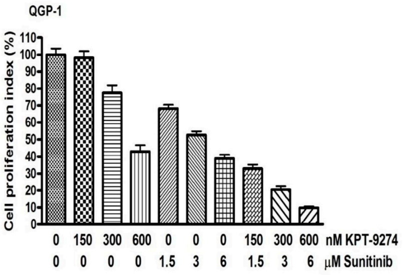

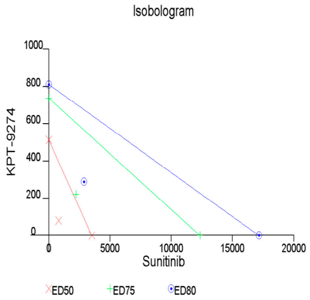

CI For experimental values

KPT-9274	Sunitinib	CI
150	1500	0.421
300	3000	0.556
600	6000	0.698
(nM)	(nM)	(nM)

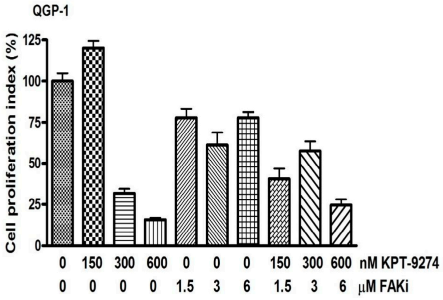

CI For experimental values

KPT-9274	FAKi	CI
150	1500	0.492
300	3000	2.498
600	6000	1.410
(nM)	(nM)	(nM)

Figure S2. KPT-9274 synergy analysis. QGP-1 cell were grown in 96 well plate at a density of 5000 cells per well overnight and exposed to indicated concentrations of either KPT-9274 in the absence or presence of equimolar ratio concentrations of sunitinib or FAKi/Defactinib (VS-6063, PF-04554878) for 72 hrs. MTT assay was performed according to standard procedure. Raw data was analyzed for synergy using CalcuSyn software. Graphs were plotted using GraphPad Prism software.

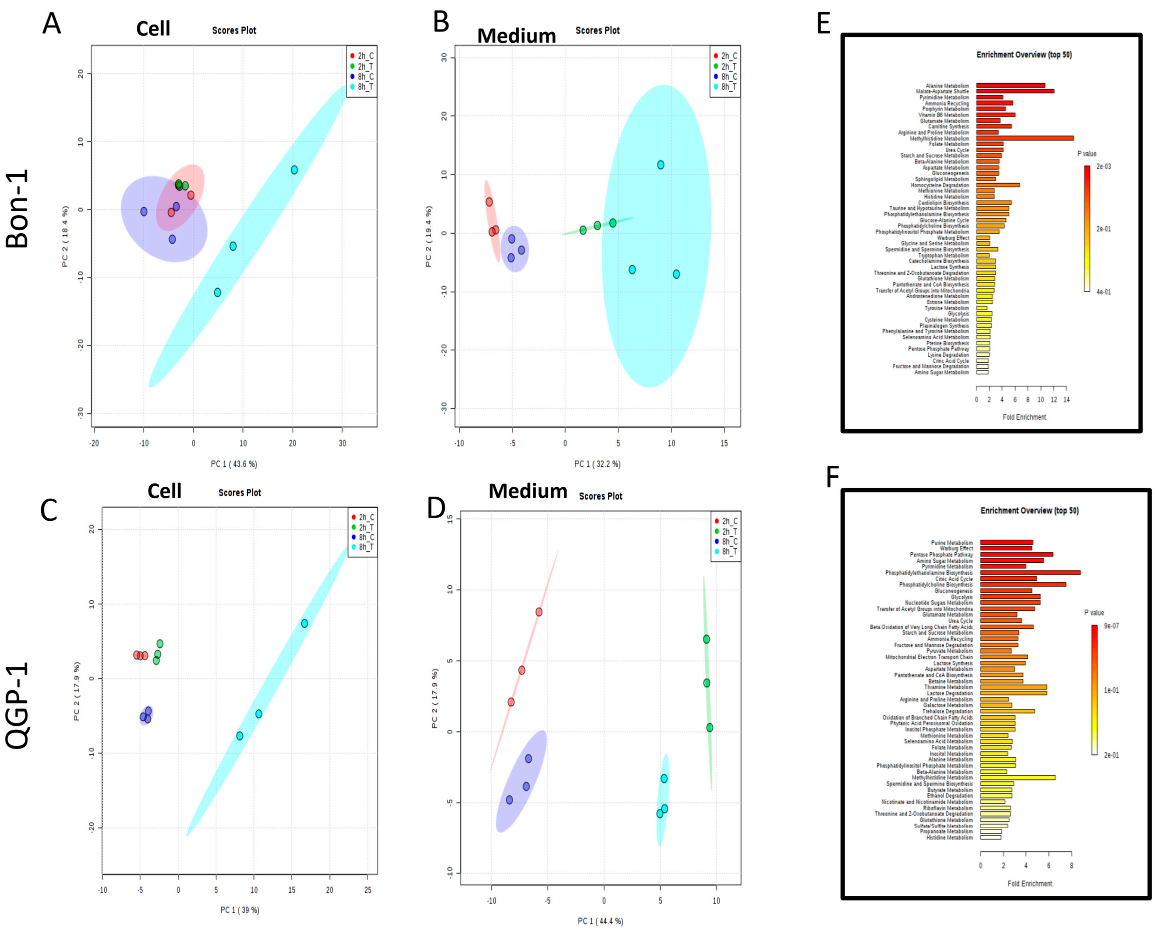

Figure S3. Targeting PAK4-NAMPT Axis alters the metabolism of PNET cells. Principal component analysis of different treatment conditions between control 2 h KPT-9274 treated 2 h for cell or control 8 h and KPT-9274 treated 8 h for Bon-1 cell and medium (**A**,**B**) and QGP1 cell and medium (**C**,**D**). Enrichment analysis for pathways altered post treatment in Bon-1 cells (**E**) and QGP-1 cells (**F**) respectively.

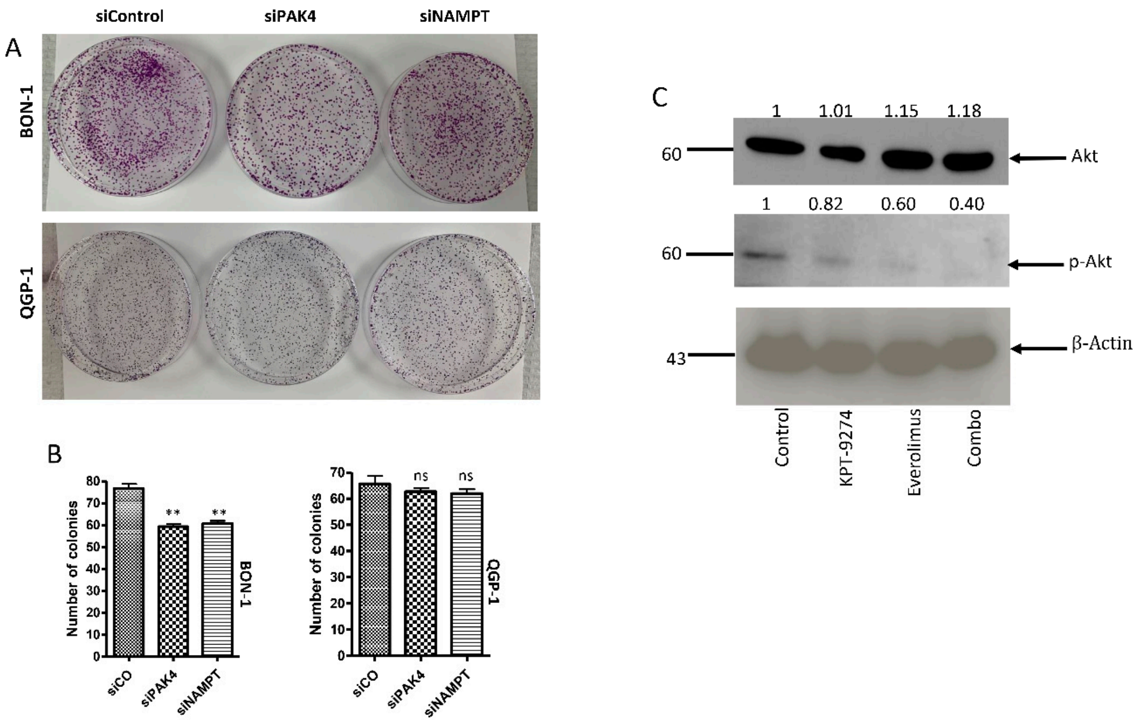

Figure S4. Targeting PAK4-NAMPT axis suppresses PNET tumor growth. (**A**) Colony formation post single siPAK4 and single siNAMPT. (**B**) Graphical representation of number of colonies. * $p < 0.05$; ** $p < 0.01$ siPAK4 $p = 0.008$; siNAMPT $p = 0.0025$. **(C)** Western blot analyses of proteins extracted from QGP-1 post treatment with 600 nM of KPT-9274, 6uM everolimus and combination.

Figure 5F full blot

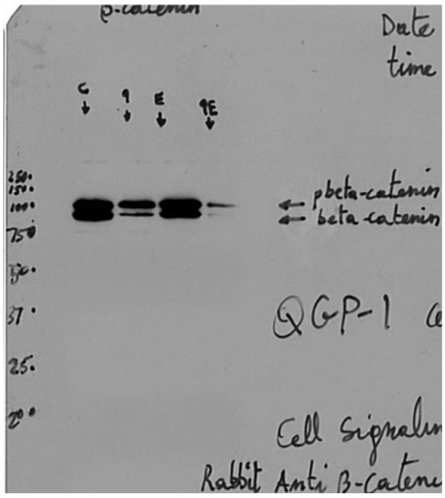

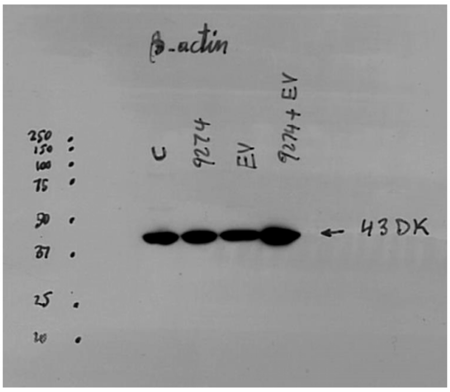

Relative protein expression (Normalized to β-actin

	Control	KPT-9274	Everolimus	Combo
Phospho β-catenin	1	0.88	0.87	0.41
β-catenin	1	0.63	0.93	0.06

Figure 4E full blot

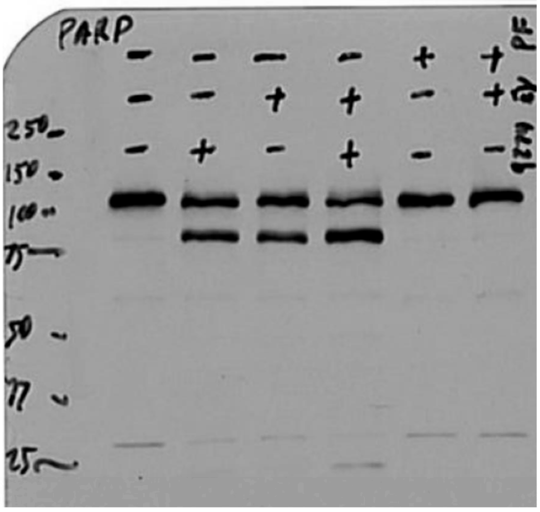

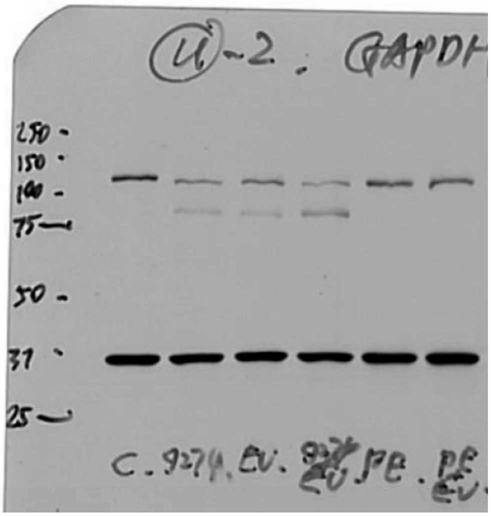

Relative protein expression (Normalized to β-actin)

	Control	9274	EV	9274 + Ev	PF	PF + Ev
PARP	1	0.89	1.12	0.78	1.26	0.84
CLEAVED PARP	0.03	0.45	0.41	0.77	0.02	0.02

Figure 1C full blot

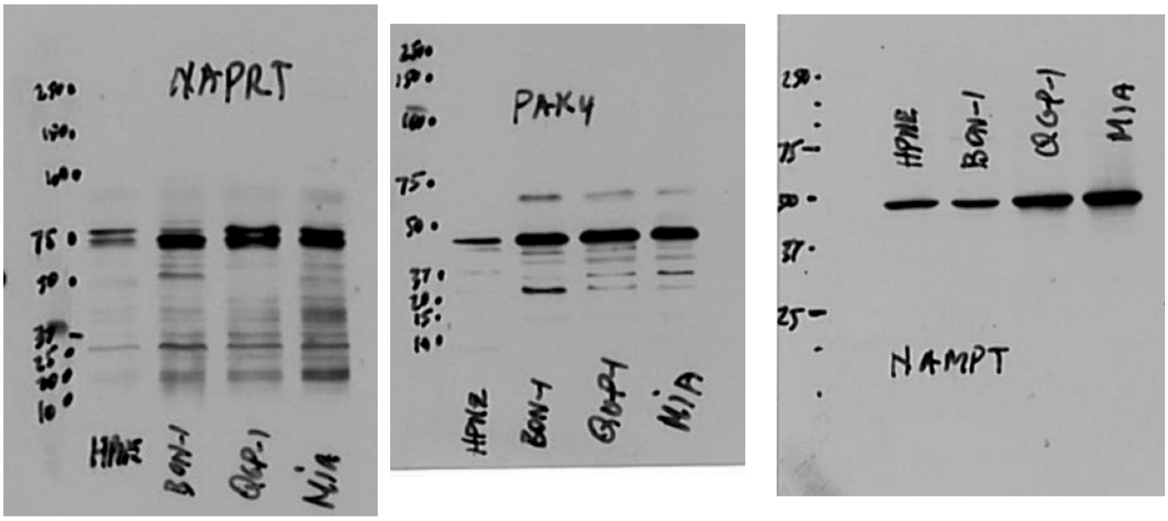

Base line expression loading control (beta-Actin and GAPDH

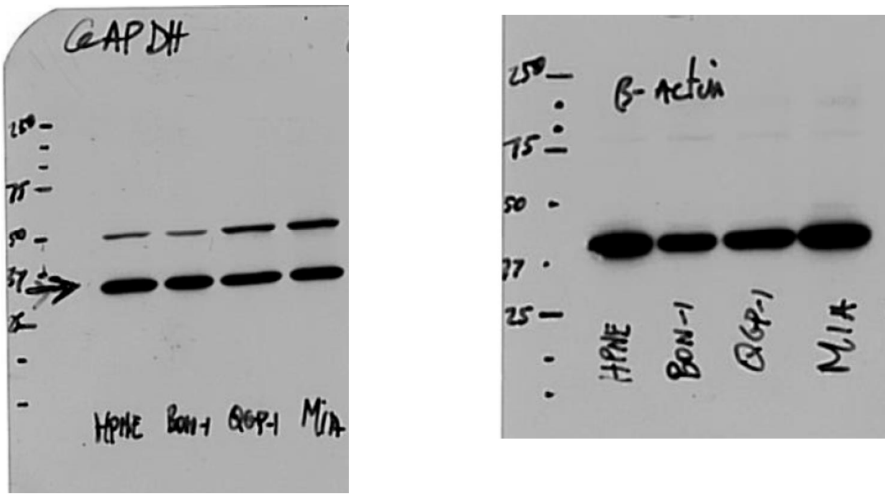

Relative protein expression (Normalized to β-actin)

	HPNE	BON-1	QGP-1	MiaPaca-2
PAK4	0.46	1.08	1.01	0.81
NAMPT	0.73	0.89	1.05	0.92
NAPRT	0.30	0.83	0.71	0.61

Supplemental figure 4C full blot

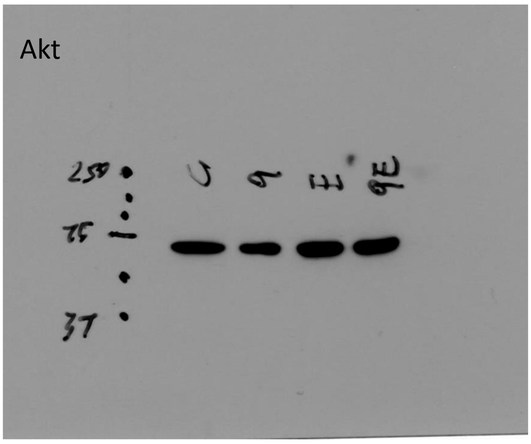

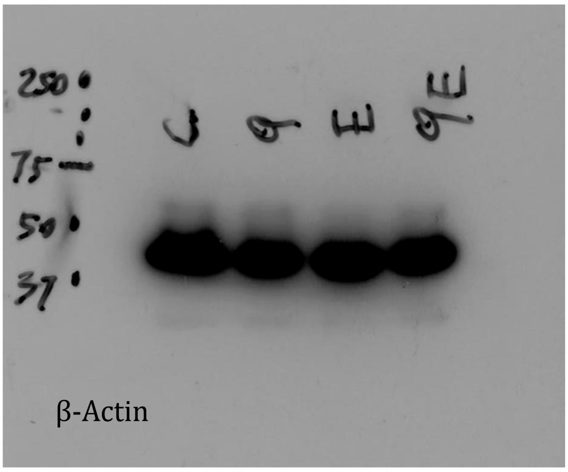

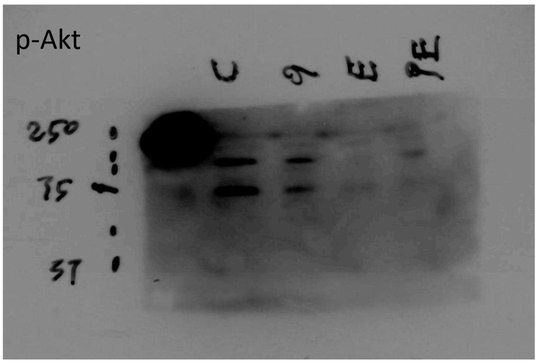

Control	KPT-9274	Everolimus	Combo
Akt	1	1.1	1.18
P-Akt	1	0.60	0.40
